# Supplementary material for: Vitamin E Deficiency Disrupts Gene Expression Networks during Zebrafish Development
Source: Nutrients. 2021 Jan 30;13(2):468. doi: 10.3390/nu13020468 (PMC7912379; doi:10.3390/nu13020468)
Supplement: Supplementary file 1 [file nutrients-13-00468-s001.zip › Supplementary:Appendix Files/Supplementary info.docx]

**Western blot antibody information**

25 μg were subjected to SDS-PAGE and processed by immunoblotting using antibody to monoclonal mouse anti-β-actin (MP Biochemicals #08691), rabbit anti-Raptor (Cell Signaling Technology #2280), rabbit anti-Rps6k (Cell Signaling Technology #2217), rabbit anti-p-Rps6k (Cell Signaling Technology #2211), rabbit anti-Eif2α (Cell Signaling Technology #9722), rabbit anti-p-Eif2α (Cell Signaling Technology #9721), rabbit anti-4E-BP1 (Cell Signaling Technology #9644), rabbit anti-p-4E-BP1 (Cell Signaling Technology #2855). The proteins were visualized by SuperSignal™ West Pico PLUS Chemiluminescent Substrate (ThermoFisher) and quantified using Bio-Rad Image System (USA).

**Supplementary Figure 1. Multi-dimensional scaling plots of E+ and E– log_10_CPM clustered at each time point prior to data filtration and normalization.**

**
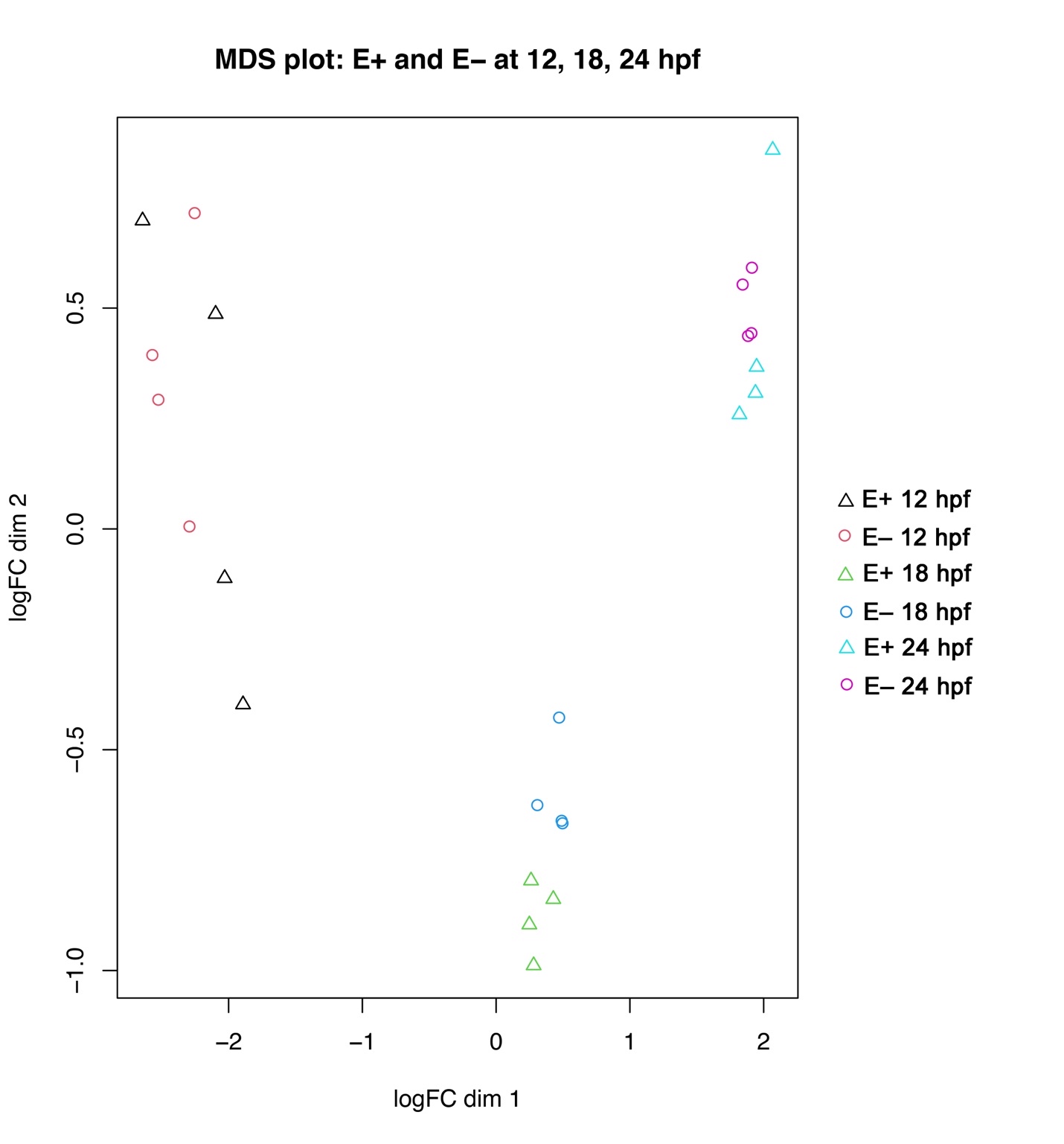
**
