# Supplementary figures and images for: Vitamin E Deficiency Disrupts Gene Expression Networks during Zebrafish Development
Source: Nutrients. 2021 Jan 30;13(2):468. doi: 10.3390/nu13020468 (PMC7912379; doi:10.3390/nu13020468)

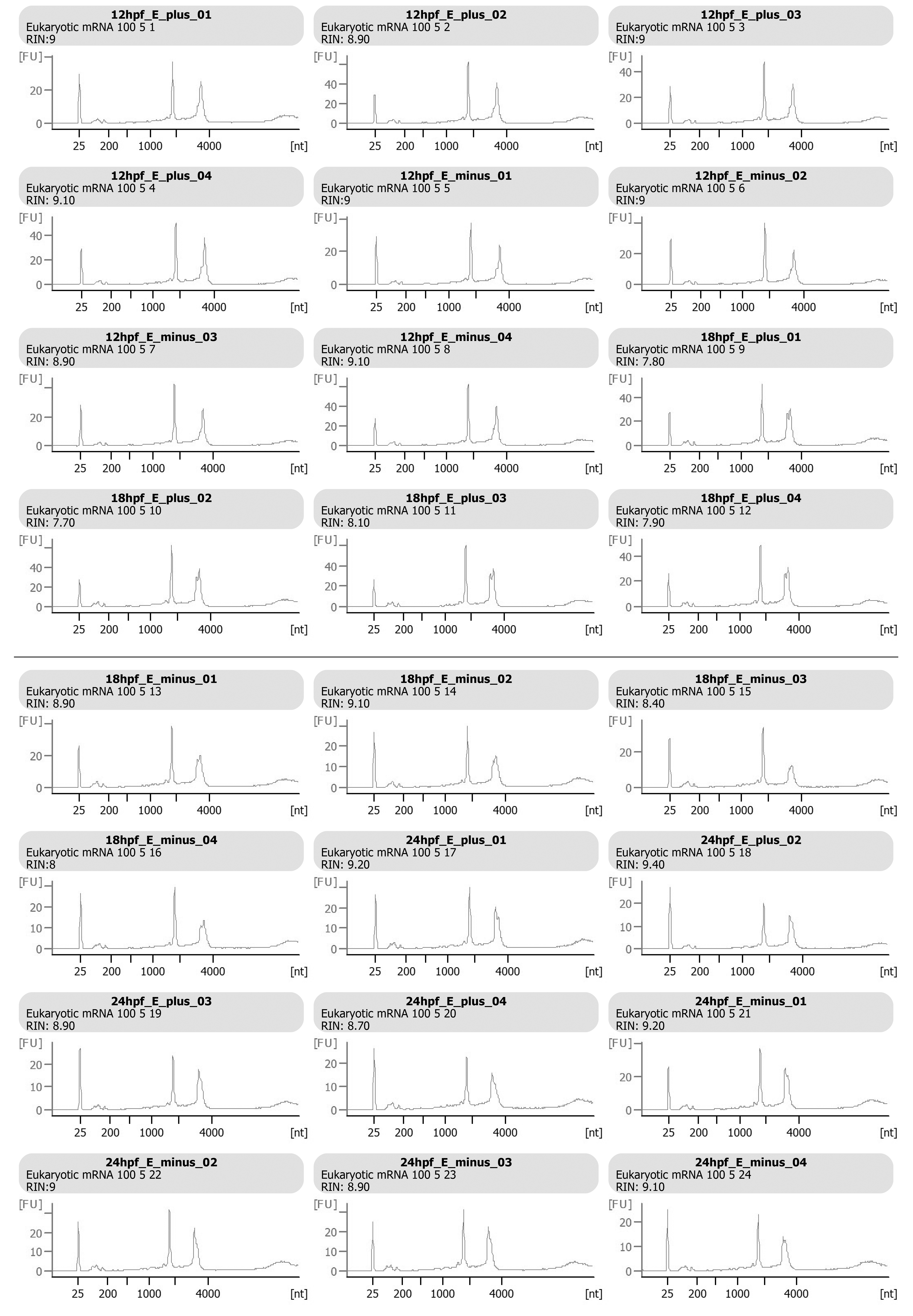

Supplement: Supplementary file 1 [file nutrients-13-00468-s001.zip › Supplementary:Appendix Files/Appendix_RIN.jpg]
